# Neoadjuvant Chemotherapy in Muscle-Invasive Bladder Cancer: A Nationwide Analysis of Eligibility, Utilization, and Outcomes

**DOI:** 10.3390/cancers17030505

**Published:** 2025-02-03

**Authors:** Ilkka Nikulainen, Antti P. Salminen, Mikael Högerman, Heikki Seikkula, Peter J. Boström, The Finnish National Cystectomy Database Research Group

**Affiliations:** 1 Department of Urology, University of Turku and Turku University Hospital, 20521 Turku, Finland; antti.salminen@tyks.fi (A.P.S.); mikael.hogerman@tyks.fi (M.H.); peter.bostrom@tyks.fi (P.J.B.); 2 Department of Surgery, Division of Urology, Central Hospital of Jyväskylä, 40620 Jyväskylä, Finland; heikki.seikkula@hyvaks.fi

**Keywords:** bladder cancer, radical cystectomy, neoadjuvant chemotherapy, NAC, muscle-invasive bladder cancer

## Abstract

Objectives: To investigate neoadjuvant chemotherapy (NAC) eligibility, utilization, and survival outcomes for muscle-invasive bladder cancer patients undergoing radical cystectomy (RC) in a Finnish population. Materials and Methods: Data from the Finnish National Cystectomy Database (2005–2017) was combined with Finnish Cancer Registry survival data. NAC utilization rates were reported, and downstaging rates were calculated based on final pathological staging. Logistic regression analyzed NAC usage and complete response (CR) predictors. Results: Since 2011, 29% of 1157 patients received NAC. Its usage remained consistent, and the number of eligible patients not receiving NAC decreased during the study period. Among NAC patients, pathology T-category was pT0 (34%), pT1-Ta-Tis (16%), pT2 (23%), pT3 (20%), and pT4 (7%) tumors, with pN0 in 82%. In the RC + NAC group, the 5-year overall survival (OS) rates were 89% for patients with no residual disease (pT0N0), 82% for those with organ-confined residual disease (pT1, Tis, Ta, T2/N0), and 49% for patients with non-organ-confined residual disease (pT3+/N+). The corresponding cancer-specific survival (CSS) rates were 93%, 86%, and 57%, respectively. Patients with organ-confined residual disease after NAC had survival outcomes comparable to those who underwent RC alone. Higher age; odds ratio (OR) 0.93, [95% Confidence Interval (CI): 0.90–0.95] and Charlson Co-morbidity Index–score [OR 0.88 (0.79–0.98)] reduced the likelihood of receiving NAC, while a smaller center size increased the probability [OR 1.82 (1.02–3.28)]. More treatment cycles [OR 0.70, (95% CI: 0.51–0.93)] and a favorable GFR [OR 0.38 (0.16–0.88)] were associated with achieving CR. Conclusion: We report that NAC is well-utilized across Finland, with CR rates comparable to recent trials. Additionally, our survival rates are reasonable, and even with organ-confined residual disease after NAC, survival outcomes are similar to those who underwent RC alone.

## Simple Summary

Muscle-invasive bladder cancer often requires a combination of treatments, including chemotherapy before surgery (neoadjuvant chemotherapy, NAC), to improve survival. In this study, we analyzed the use and effectiveness of NAC given before bladder removal surgery in Finland. We aimed to understand how often this treatment is used, how well it works, and which patients benefit most. We found that about one-third of patients received NAC, with usage rates staying steady over the years. Patients who received the treatment often showed reduced tumor size, and their overall survival rates were better than those who had surgery alone. Factors like older age and poor health reduced the likelihood of receiving NAC, while better kidney function and more chemotherapy cycles improved outcomes. These findings support the effective use of chemotherapy before surgery and highlight the importance of selecting the right patients for this approach.

## 1. Introduction

Bladder cancer (BC) is the seventh most common cancer in men globally, with approximately 25% of newly diagnosed cases being muscle-invasive (MIBC). MIBC is associated with substantial morbidity and mortality, and radical cystectomy (RC) remains the standard treatment. Neoadjuvant chemotherapy (NAC) is recommended for eligible patients with MIBC undergoing RC [1]. A median improved survival of over 30 months with platinum-based NAC [2] along with a 5–8% overall survival (OS) benefit has been confirmed in 4 meta-analyses [3,4,5,6]. The rationale for NAC lies in administering chemotherapy at an early stage, when the burden of micrometastases is presumed to be minimal and when patients are more likely to tolerate the treatment. This early administration, unlike with adjuvant treatment, enables an assessment of tumor response to chemotherapy, and in advanced cases of NAC, may also downstage the tumor and potentially facilitate a more successful surgical resection [1,7].

While NAC has been demonstrated to enhance survival rates, the regimen remains a topic of ongoing debate. Since the introduction of NAC for MIBC in Finland in 2008, gemcitabine-cisplatin (GC) has become the predominant regimen, applied nationally for patients with adequate performance status (PS 0–1), sufficient kidney function (glomerular filtration rate, GFR > 50–60 mL/min), and no significant co-morbidities. Depending on the center preferences, GC is administered in three to four cycles prior to RC with three-week interval between the cycles. Finland’s public healthcare system ensures equal access to care for all patients. As demonstrated in our previous research, NAC has been consistently utilized across academic and non-academic centers throughout the country, with comparable survival outcomes [8]. Nevertheless, it is worth noting that GC is not the standard regimen in all countries and that all pivotal randomized clinical trials utilized regimens other than GC [6,9]. Globally, the methotrexate–vinblastine–doxorubicin–cisplatin (MVAC) regimen is also widely used. Research findings indicate that MVAC treatment, while potentially more effective, also tends to be associated with higher levels of toxicity [10].

Recently, novel immunotherapies have emerged as promising treatment options for MIBC. Immune checkpoint inhibitors, such as PD-1/PD-L1 inhibitors, have shown encouraging results in clinical trials, both as monotherapy and in combination with chemotherapy. These immunotherapies are being investigated in various settings, including neoadjuvant and adjuvant treatments, potentially offering new strategies to improve outcomes for MIBC patients [11,12,13,14,15,16].

The pathological staging using the TNM classification [17] after surgery is the leading prognostic indicator of survival. The survival benefit of NAC is linked to tumor downstaging, which likely indicates a positive response against potential micrometastases [18]. Despite established efficacy of NAC, it is still widely underutilized in clinical practice with implementation rates of 20–30%. The most common reasons for the omission or discontinuation of NAC are contraindications, lack of tolerance, or disease progression. [19] This is frequently observed in patients who possess a range of co-morbidities, have advanced age, or are frail [20]. Moreover, platinum-based chemotherapy can cause adverse effects (AE) such as decreased renal or kidney function, hearing loss, and myelosuppression [20,21]. If a patient exhibits any of these predisposing conditions, NAC should be administered cautiously.

Our most comprehensive national study to date aimed to assess the real-world effectiveness of GC as a NAC regimen. Additionally, we aimed to evaluate patient eligibility, the utilization rates, downstaging rates, and survival outcomes of patients who received NAC in a population-based context.

## 2. Materials and Methods

The Finnish National Cystectomy Database (FNCD) is an online platform initiated by academic urologists and was used for this retrospective study. The FNCD, developed by academic urologists exclusively for research purposes, was designed to be secure and accessible via the Internet. The database has been thoroughly described in previous publications [8]. As a comprehensive data repository, it includes information from all 16 hospitals in Finland where RCs took place from 2005 to 2017, covering the period before the national centralization of RCs in 2018.

The study’s inclusion criteria mandated that the RC was conducted for BC. Collected data included patient characteristics, tumor, and treatment details. For patients receiving NAC from 2011 to 2017, collected data included the chemotherapy regimen, number of cycles, pathological response, and possible AEs related to NAC. The year 2011 was chosen as there was a national, widespread acceptance and increased usage of NAC in 2011. The final pathological T-stage of the RC specimen was recorded and used to evaluate tumor downstaging.

In assessing eligibility for NAC, we defined patients as “NAC eligible” based on the following criteria supported by literature and national oncological guidelines: age ≤ 75 years, TUR-BT T-category ≥ T2, and a GFR ≥ 60 mL/min [22,23]. A bar chart was used to illustrate the number and percentage of patients categorized as receiving NAC, being eligible but not receiving it, and being ineligible.

Associations between NAC administration and explanatory variables (age, Body Mass Index (BMI), gender, smoking status, Charlson Co-morbidity Index (CCI-) score, ASA class, center size, modality, number of NAC cycles, TURB-T, and pT category) were summarized with descriptive statistics. The normality of variables was evaluated visually and with the Shapiro–Wilk test. Due to the non-normality of the continuous variables, nonparametric methods were used. The association between explanatory variables and the probability of having a CR (pT0) and receiving NAC was studied with a multivariate logistic regression model and reported with odds ratios (OR). Explanatory variables included age, CCI score, center size, BMI, number of NAC cycles, and GFR.

As previously reported, survival data were obtained from the Finnish Cancer Registry (FCR) [8]. Kaplan–Meier plots were used to assess both OS and cancer-specific survival (CSS) for all patients recorded in the database. Survival duration was calculated starting from the date of RC until death, the last known follow-up visit, or the final follow-up date, which was set at 2018. The survival graphs were categorized based on the final pathological TNM classification, grouping the patients into no residual disease (pT0N0), organ-confined residual disease (pTa, Tis, T1, T2/N0), and non-organ-confined residual disease (pT3+/N+) groups. Statistical analyses involved the use of the univariate log-rank test for comparing survival among patient subgroups, with hazard ratios (HR) also calculated. The statistical significance level was set at 0.05 in all tests (two-tailed), and 95% confidence intervals (CIs) were calculated. Analyses were performed using RStudio, version 2023.09.1, based on R, version 4.2.2.

## 3. Results

**Figure 1:**
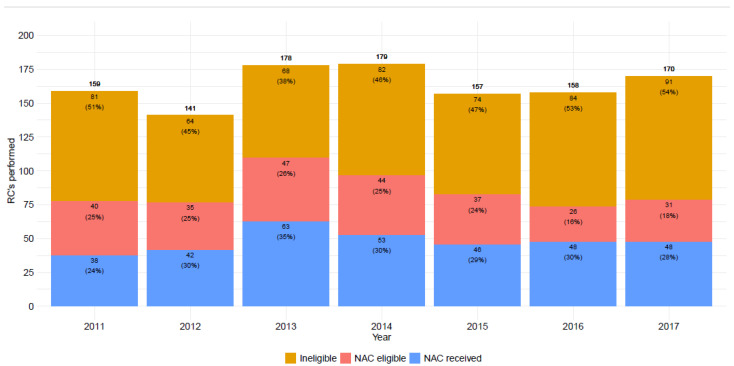
Radical cystectomy rates, neoadjuvant chemotherapy (NAC) eligible patients, and NAC utilization 2011–2017.

The basic characteristics of the study population are presented in Table 1, and Figure 1 illustrates RC rate and NAC utilization from 2011 to 2017. During this period, 338 out of 1157 patients (29%) received NAC. The utilization rate remained consistent throughout the study period, ranging from 24 to 35%. Approximately half of the patients were ineligible for NAC at the time of RC, while the proportion of NAC-eligible patients not receiving NAC declined over the study period, dropping from 25% to 16–18%. The median age of patients receiving NAC was 65 years, and 278 (82%) were men. Ninety-five percent of the patients receiving NAC had an ASA class of 2 or 3. The most commonly used NAC regimen was GC in 298 (92%) patients, while 21 (6%) patients received carboplatin-gemcitabine (CAR-GEM). Of the patients receiving NAC, 80% received three or four cycles, while 19% received two or less, and 1% of the patients received five or six cycles as induction chemotherapy before surgery. Most tumors among NAC recipients were muscle invasive (88%) in the TURB-T specimen. At cystectomy, pT0 status was recorded in 34% and 15% among those receiving and not receiving NAC, respectively. In contrast, non-organ-confined tumors (pT3-4) were more common (44%) in patients not receiving NAC than in those receiving it (26%). Lymph nodes positive for cancer were detected in 15% and 24% of patients receiving and not receiving NAC, respectively.

The factors affecting the probability of NAC and CR are presented in Table 2. Increasing patient age [OR 0.93, 95% confidence interval (CI): 0.90–0.95] and a higher CCI score [OR 0.88 (0.79–0.98)] were associated with a reduced risk of receiving NAC. However, a lower volume center size was linked to a higher risk of receiving NAC [OR 1.82 (1.02–3.28)]. Additionally, in the logistic regression focusing on patients who received NAC, the number of treatment cycles [OR 0.70 (0.51–0.93)] and a favorable GFR [OR 0.38 (0.16–0.88)] were in relation to achieving a CR (pT0) in the final pathological T-category.

**Figure 2:**
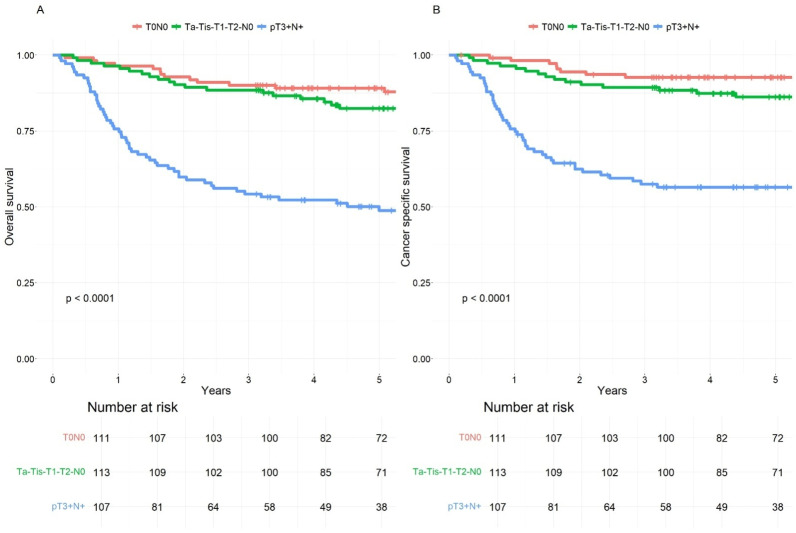
(**A**). Overall survival of neoadjuvant chemotherapy (NAC) + radical cystectomy (RC) patients according to pT category. (**B**). Cancer-specific survival of NAC + RC patients according to pT category.

**Figure 3:**
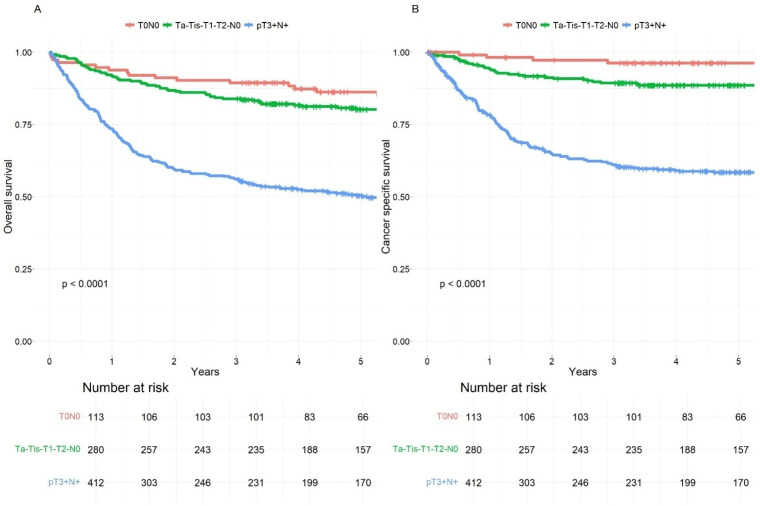
(**A**). Overall survival of radical cystectomy (RC) only patients according to pT category. (**B**). Cancer-specific survival of RC-only patients according to pT category.

In Table 3 we compared the 5-year OS and CSS rates between patients who underwent RC with NAC and those who had RC only, stratified by the pT category. The median follow-up time in the study was 5.6 years (IQR: 2.8–7.8 years). The findings indicate that patients with a non-organ-confined tumor had significantly lower survival rates, with higher hazard ratios for death, compared to those with no residual tumor or an organ-confined residual tumor. Notably, RC + NAC patients generally had slightly better overall survival outcomes across pT0-pT2 categories compared to RC-only patients. Nonetheless, patients with tumor growth beyond the bladder wall at the time of RC experienced significantly poorer survival outcomes, particularly evident among those who had undergone NAC. (Figure 2 and Figure 3).

**Figure A1:**
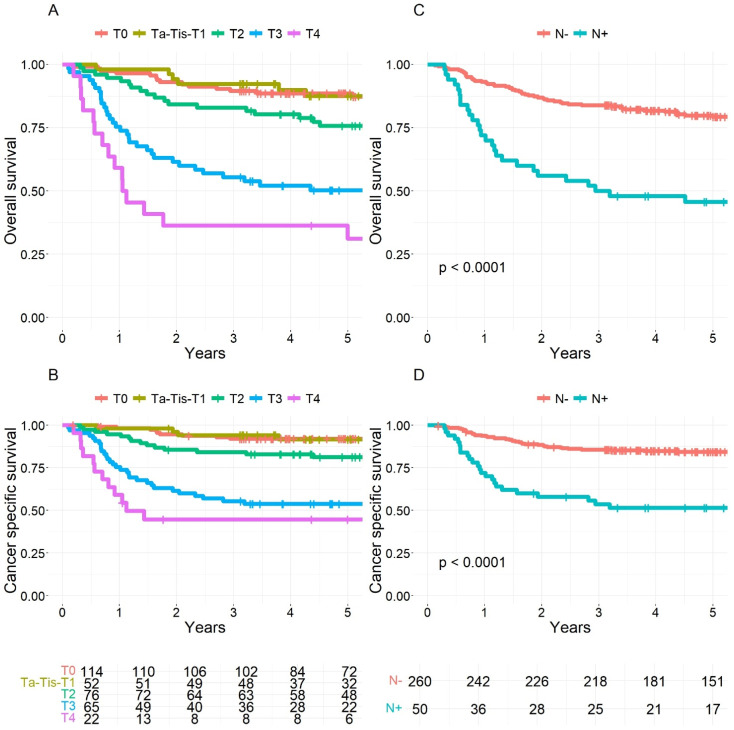
(**A**). Overall survival of neoadjuvant chemotherapy (NAC) + radical cystectomy (RC) patients according to pT category. (**B**). Cancer-specific survival of NAC + RC patients according to pT category. (**C**). Overall survival of NAC + RC patients according to pN category. (**D**). Cancer-specific survival of NAC + RC patients according to pN category.

**Figure A2:**
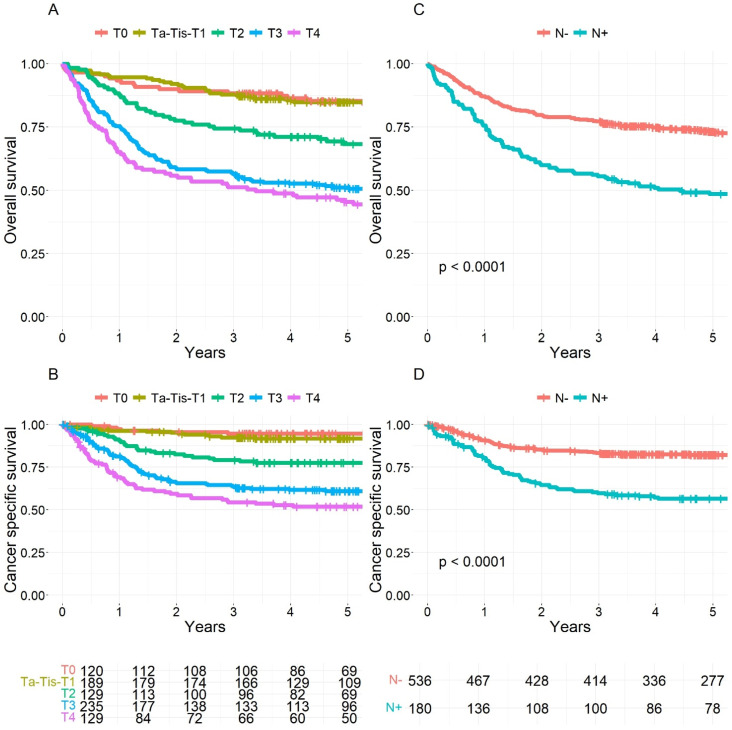
(**A**). Overall survival of radical cystectomy (RC)-only patients according to pT category. (**B**). Cancer-specific survival of RC-only patients according to pT category. (**C**). Overall survival of RC-only patients according to pN category. (**D**). Cancer-specific survival of RC-only patients according to pN category.

In the Appendix A Table A1, we compared the 5-year OS and CSS rates between patients who underwent RC with NAC and those who had RC only, stratified by pT and pN category. The findings indicate that patients with more advanced tumor stages (pT3, pT4) or lymph node involvement (pN+) had significantly lower survival rates, with higher hazard ratios for death, compared to those with less advanced disease (pT0-T2, pN0). Notably, RC + NAC patients generally had slightly better survival outcomes across pT0-pT2 categories, particularly in OS, compared to RC-only patients. Nonetheless, patients with tumor growth beyond the bladder wall at the time of RC experienced significantly poorer survival outcomes, particularly evident among those who had undergone NAC (Appendix A Figure A1 and Figure A2).

**Figure A3:**
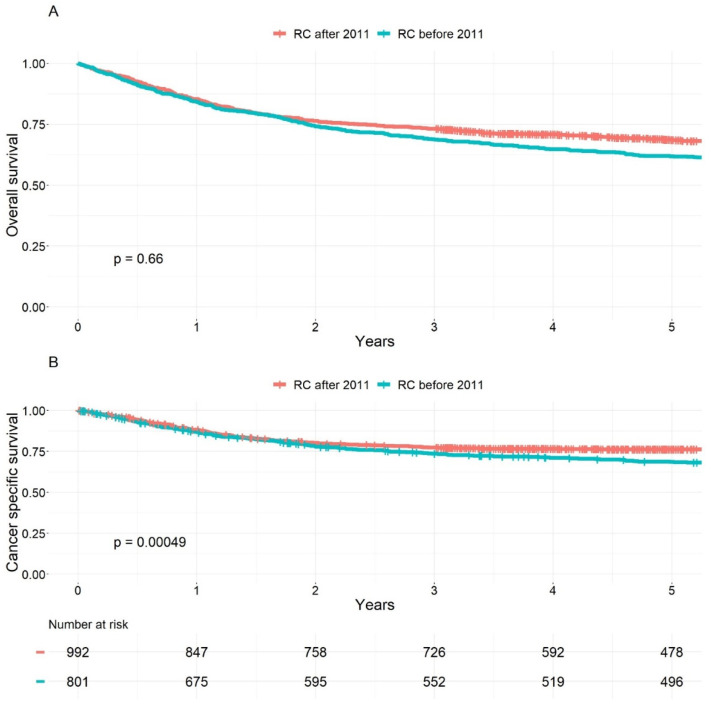
(**A**). Overall survival of the study population before and after 2011. (**B**). Cancer-specific survival of the study population before and after 2011.

In the Appendix A Table A2, we compared the 5-year OS and CSS rates for patients undergoing surgery before and after 2011 to evaluate the survival impact of increased NAC utilization. Prior to 2011, the 5-year OS was 62%, and the CSS was 69%. After 2011, the 5-year OS improved to 69%, and the CSS to 77%. The improvement was statistically significant for CSS, with HR [OS: 0.97 (0.83–1.12), CSS: 1.37 (1.15–1.64)]. This is illustrated in Appendix A Figure A3.

## 4. Discussion

The main findings of our study highlight the beneficial impact of NAC on tumor downstaging and emphasize factors influencing its administration. In Finland, NAC was first introduced in 2008 and initially limited to a single center until 2010. Since 2011, NAC gained nationwide acceptance, with a utilization rate of 29%, which remained consistent throughout the study period, while the number of untreated NAC-eligible patients decreased. The study revealed that 34% of patients achieved a complete response (pT0), while 16% had a partial response (PR, pTa-T1-Tis). Half of the patients had muscle-invasive or locally advanced residual disease (pT2-4) at the time of RC, but those who received NAC had significantly lower tumor stages than those who did not. The risk of receiving NAC decreased with higher age and co-morbidities, as indicated by the CCI score, while lower-volume centers were more likely to administer NAC. Importantly, a higher number of NAC cycles (three or more) and a better kidney function were statistically associated with an increased likelihood of achieving a pT0 in the RC specimen.

The main survival advantage of NAC is linked to tumor downstaging [18]. Patients with CR (pT0) have the best survival outcomes, and patients with PR (pTis-Ta-T1) also do well. However, previous studies have suggested that non-NAC-responders (pT2-4) have the worst survival outcomes [24,25]. The reported CR rates in the literature using platinum-based chemotherapy have been approximately 25% for both MVAC and GC [24]. It should be noted that a thorough TUR-BT also contributes to cases of pT0 in patients undergoing RC, with reported rates ranging up to 20% [26,27]. Our study had comparable results, with a pT0 rate of 15% observed in patients who did not receive NAC. The prospective VESPER trial compared MVAC and GC in a NAC setting, and organ-confined response (<pT3N0) was observed in favor of MVAC (77% vs. 63%). The MVAC treatment group also demonstrated a significantly longer time to progression compared to GC, albeit with a higher incidence of adverse events [10]. Our study reports a CR rate of 34%, and organ-confined response was observed in 73% of our patients in the GC arm, suggesting that GC works well in a real-world setting. In addition, our CR patients did extremely well with a 5-year CSS of 93%, as did patients with organ-confined residual disease (CSS 86%). This being said, 27% had extravesical tumor growth (T3–T4) despite receiving NAC and had worse prognosis as NAC only delayed their surgery. However, the 5-year OS for these patients was still 49%, in comparison to 50% in the RC-only population. The rather small difference in survival rates is likely due to the restricted timeframe of our survival data, which only covers until 2018, and since the administration of NAC began in 2011, the follow-up period may be relatively short for certain patients.

Patients with non-organ-confined residual disease have the poorest survival outcomes, highlighting one of the challenges of NAC: determining which patients benefit from the treatment and which tumors respond. Different imaging modalities, such as MRI of the bladder with the VI-RADS classification have shown promise in assessing tumor response to NAC, but results are still somewhat conflicting [28,29,30]. Genomic biomarkers have been reported to associate with response to NAC to find patients who would benefit the most [31]. Identifying patients who are likely to respond well to NAC would be particularly beneficial for those considering bladder-preserving treatment options or who are ineligible for RC [32]. However, it is worth noting that while these biomarkers have shown promise in predicting NAC response, more research is needed to validate their use in clinical practice. One of these validation studies is also ongoing in the Nordic countries (NorCys I-trial, NCT04523025).

In previous clinical trials, the utilization rate of NAC varied from 20% to 30% [19]. Our study shows a similar rate of 29% nationwide in Finland. This utilization rate precedes centralization efforts and was observed even in low- and medium-volume centers (less than 20 RCs annually). This is likely due to differences in patient characteristics between centers, as low-volume centers may manage a higher proportion of patients with fewer co-morbidities and better PS, making them more suitable candidates for NAC. GC is the main modality used, but some centers also used CAR-GEM, which is the chemotherapy treatment of choice for cisplatin-ineligible patients with metastatic disease but is not recommended for NAC by any guidelines [1]. One of the biggest issues regarding platinum-based chemotherapy, and the main reason why utilization rates are fairly low, is that nearly 50% of BC patients are ineligible due to co-morbidities, mainly due to poor kidney function and PS [33]. A similar observation was made in a study concerning NAC eligibility among BC patients in Denmark [23], and these findings align with the results of our study (Figure 1). Moreover, we identified a negative association between a higher CCI score and older patient age and the risk of receiving NAC. Both of these characteristics likely contribute to increased frailty, which is why the use of NAC is avoided. There are limited treatment options available for these patients, but a recent example is the use of split-dose GC, which has demonstrated that cisplatin can be a viable treatment choice for select patients with a GFR of less than 60 mL/min [34]. Immune checkpoint inhibitors have emerged as promising alternative treatments in the neoadjuvant setting. Phase II trials using single-agent immunotherapy have shown encouraging results, with pembrolizumab achieving 42% complete pathological remission (pT0) in the PURE-01 trial, while atezolizumab demonstrated a 31% pathologic CR rate in the ABACUS trial [12,13]. Combination immunotherapies have also shown promise [14,15]. Furthermore, the NIAGARA study group recently reported groundbreaking findings, showing that perioperative durvalumab added to cisplatin-based NAC significantly improved both event-free survival and overall survival compared to NAC alone (2-year OS: 82% vs. 75%) [11]. However, the EAU guidelines state that neoadjuvant immunotherapy should only be administered in a clinical trial setting due to the lack of data concerning long-term survival benefit. The only use for immunotherapy currently endorsed by the guidelines is adjuvant nivolumab if the patient has a significant residual tumor in the final pathological report despite having received platinum-based chemotherapy [1,16].

Additionally, in the VESPER trial, a comparison of toxicity and tolerance between MVAC and GC was conducted. Among the NAC group, 218 patients received dd-MVAC while 219 received GC. Notably, the study reported an impressive 84% of patients completing four full cycles of GC, with only 16% receiving fewer cycles [20]. In our study, 80% of NAC recipients achieved the recommended number of GC cycles (three or four), and a higher number of cycles administered during NAC increased the probability of achieving a pT0 in the RC specimen (OR 0.70). Furthermore, a higher GFR value was also linked to an increased likelihood of achieving a pT0 (OR 0.38), likely because patients with better GFR are able to tolerate more treatment cycles. In the VESPER trial, 55% of GC patients had at least one grade three hematological toxicity; mostly neutropenia was reported (46%). In addition, 5–7% of patients had grade three non-hematological toxicities, including kidney (5%), liver (5%), and cardiovascular (7%) toxicity events. One NAC-related death was reported in the GC arm and three in the dd-MVAC arm [20]. AEs in our study population have been reported by Salminen et al. in 2019 [35], and 54% of patients had some form of AE. A Swedish group, however, reported that 96% of patients experienced any grade AEs during NAC [36], suggesting that AEs are underreported in the literature.

Although our study demonstrated a small survival benefit with increased NAC utilization rates in patients who underwent surgery after 2011, as well as impressive CR rates in the aforementioned patients, it is important to acknowledge the limitations commonly associated with retrospective studies. These include potential biases such as selection bias to treatment and patients lost to follow-up. The selection bias results in healthier patients generally receiving NAC, leading to improved survival outcomes. Additionally, the median follow-up duration was 5.6 years, with follow-up continuing until 2018, leading to a relatively short follow-up period for some patients, as previously mentioned. Unfortunately, we lacked data on the cT-stage of the patients and instead relied on the T-category from the TUR-BT specimen. In the RC + NAC group, 12% of patients had NMIBC pathology in their TUR-BT specimens. This reflects cases where tumors were clearly muscle-invasive on imaging, but only superficial samples were resected during TUR-BT. Despite these limitations, the study results were strengthened by our thorough data covering every RC performed nationwide during the study period. The large number of patients included and the validation of follow-up data from various centers in combination with the FCR data helped to reduce the risk of underreporting mortality or cause of death.

## 5. Conclusions

The utilization of NAC with GC in MIBC patients has remained steady in Finland. Our study demonstrates that GC use is linked to higher rates of pathological downstaging and improved overall survival compared to RC-only patients. A key finding of our study is that patients who received NAC and had residual organ-confined disease (<pT3, N0) showed survival outcomes comparable to those who underwent RC alone. Frailty (older age and higher CCI) reduces the likelihood of receiving NAC, while smaller center size increases the probability. Additionally, adequate kidney function likely enables patients to receive more NAC cycles, increasing the chances of achieving downstaging. These findings align with previous studies, highlighting the importance of patient selection to optimize outcomes and supporting the use of GC as NAC.

## Figures and Tables

**Table 1:** The basic characteristics and tumor data of the patients undergoing radical cystectomy (RC) in Finland from 2011 to 2017 and sub-cohorts of the patients receiving or not receiving neoadjuvant chemotherapy (NAC) before the surgery.

Characteristic	Overall *N* = 1157	RC ^1^ Only *N* = 819	RC ^1^ + NAC ^2^ *N* = 338
Age, median (IQR ^3^)	70 (63, 76)	72 (65, 77)	65 (60, 71)
BMI ^4^,median (IQR ^3^)	26 (23, 29)	26 (23, 29)	26 (23, 29)
Gender, *N* (%)			
Female	229 (20%)	169 (20%)	60 (18%)
Male	928 (80%)	650 (80%)	278 (82%)
Smoking status, *N* (%)			
Active	252 (33%)	145 (29%)	107 (41%)
Never	239 (30%)	176 (34%)	63 (23%)
Stopped	283 (37%)	185 (37%)	98 (36%)
CCI ^5^ score, median (range)	4.00 (3.00, 6.00)	4.00 (3.00, 6.00)	4.00 (3.00, 5.00)
ASA ^6^-class, *N* (%)			
1	33 (3%)	24 (3%)	9 (3%)
2	362 (34%)	219 (29%)	143 (45%)
3	597 (57%)	443 (60%)	154 (50%)
4	64 (6%)	60 (8%)	4 (1%)
Center size, *N* (%)			
Large ^7^	777 (67%)	547 (67%)	230 (68%)
Medium ^8^	306 (26%)	231 (28%)	75 (22%)
Small ^9^	75 (6.4%)	41 (5.0%)	33 (9.8%)
Modality *N* (%)			
CIS-GEM ^10^	298 (25%)	N/A ^15^	298 (92%)
CAR-GEM ^11^	21 (2%)	N/A	21 (6%)
Other	5 (<1%)	N/A	5 (2%)
Unknown	17	N/A	17
Cycles *N* (%)			
1	25 (2%)	N/A	25 (8%)
2	33 (3%)	N/A	33 (10%)
3	118 (10%)	N/A	118 (35%)
4	153 (13%)	N/A	153 (45%)
5	2 (<1%)	N/A	2 (<1%)
6	1 (<1%)	N/A	1 (<1%)
Unknown	2	N/A	2
TUR-BT ^12^ T-category			
NMIBC ^13^	338 (31%)	296 (39%)	42 (12%)
MIBC ^14^	730 (69%)	444 (61%)	286 (88%)
pT category			
T0	234 (21%)	120 (15%)	114 (34%)
Ta-Tis-T1	241 (21%)	189 (23%)	52 (16%)
T2	205 (18%)	129 (16%)	76 (23%)
T3	300 (27%)	235 (29%)	65 (20%)
T4	151 (13%)	129 (16%)	22 (7%)
pN category			
N−	796 (74%)	536 (70%)	260 (82%)
N+	230 (22%)	180 (24%)	50 (16%)
Nx	52 (5%)	47 (6%)	5 (2%)

^1^ RC = radical cystectomy; ^2^ NAC = neoadjuvant chemotherapy; ^3^ IQR = Inter Quartile Range; ^4^ BMI = Body Mass Index (kg/m^2^); ^5^ CCI = Charlson Co-morbidity Index; ^6^ ASA = American Society of Anesthesiologists Classification; ^7^ Large = over 20 RCs performed annually; ^8^ Medium = 5–20 RCs performed annually; ^9^ Small = less than 5 RCs performed annually; ^10^ CIS-GEM = cisplatin–gemcitabin; ^11^ CAR-GEM = carboplatin–gemcitabin; ^12^ TUR-BT = Transurethral Resection of Bladder Tumor; ^13^ NMIBC = non-muscle Invasive bladder cancer (pT0-Ta-Tis-T1); ^14^ MIBC = muscle invasive bladder cancer (pT2-T3-T4); ^15^ N/A = not available.

**Table 2:** Logistic regression of the probability of receiving neoadjuvant chemotherapy (NAC) and complete response (CR, pT0).

Characteristic	OR ^1^	CI ^2^ 95%	*p*-Value
Probability of receiving NAC			
Age	0.93	0.90–0.95	<0.001
CCI ^3^ score	0.88	0.80–0.98	0.019
Center size			
Large ^4^ center	Ref	Ref	Ref
Medium ^5^ center	0.85	0.58–1.27	0.44
Small ^6^ center	1.82	1.02–3.28	0.043
Probability of having a CR (pT0)			
Body Mass Index	1.02	0.99–1.09	0.36
Number of NAC cycles	0.70	0.51–0.93	0.019
GFR ^7^	0.38	0.16–0.88	0.028

^1^ OR = odds ratio; ^2^ CI = confidence interval; ^3^ CCI = Charlson Co-morbidity Index; ^4^ Large = more than 20 RCs performed annually; ^5^ Medium = 5–20 RCs performed annually; ^6^ Small = less than 5 RCs performed annually; ^7^ GFR = glomerular filtration rate.

**Table 3:** Survival rates of radical cystectomy (RC) + neoadjuvant chemotherapy (NAC) and RC-only patients according to pT category.

	RC + NAC Patients				RC-Only Patients			
Characteristic	Overall Survival		Cancer Specific Survival		Overall Survival		Cancer Specific Survival	
	5-y.	HR ^1^ (95% CI ^2^)	5-y.	HR ^1^ (95% CI ^2^)	5-y.	HR ^1^ (95% CI ^2^)	5-y.	HR ^1^ (95% CI ^2^)
pT category								
T0/N0	89%	Reference	93%	Reference	86%	Reference	96%	Reference
Ta-Tis-T1-T2/N0	82%	1.7 (0.9–3.0)	86%	1.9 (0.8–4.4)	80%	1.3 (0.8–2.0)	89%	3.3 (1.2–9.3)
T3+/N+	49%	4.9 (2.9–8.4)	57%	7.8 (3.7–16.5)	50%	3.4 (2.2–5.1)	59%	14.1 (5.2–38.1)

^1^ HR = hazard ratio; ^2^ CI = confidence interval.

## Data Availability

The data that support the findings of this study are available from the corresponding author, Ilkka Nikulainen, upon reasonable request.

## Supplementary Materials

The following supporting information can be downloaded at https://www.mdpi.com/article/10.3390/cancers17030505/s1, Supplementary File: The Finnish National Cystectomy Database Research Group researchers information.

## Appendix A

**Table A1:** Survival rates of radical cystectomy (RC) + neoadjuvant chemotherapy (NAC) and RC-only patients according to pT and pN category.

	RC + NAC Patients				RC-Only Patients			
Characteristic	Overall Survival		Cancer Specific Survival		Overall Survival		Cancer Specific Survival	
	5-y.	HR ^1^ (95% CI ^2^)	5-y.	HR ^1^ (95% CI ^2^)	5-y.	HR ^1^ (95% CI ^2^)	5-y.	HR ^1^ (95% CI ^2^)
pT category								
T0	89%	Reference	92%	Reference	85%	Reference	95%	Reference
Ta-Tis-T1	87%	1.2 (0.6–2.7)	92%	1.0 (0.3–3.1)	85%	1.1 (0.7–1.7)	92%	1.6 (0.6–4.0)
T2	75%	2.1 (1.2–3.9)	81%	2.4 (1.1–5.6)	68%	1.8 (1.1–2.9)	76%	4.8 (2.0–11.6)
T3	50%	4.4 (2.5–7.7)	54%	7.6 (3.6–16.0)	51%	3.3 (2.1–5.0)	61%	9.0 (4.0–20.7)
T4	31%	8.3 (4.3–16.1)	45%	12.0 (5.0–28.4)	45%	3.7 (2.3–5.7)	52%	12.6 (5.5–29.3)
pN category								
N−	79%	Reference	84%	Reference	73%	Reference	82%	Reference
N+	46%	3.3 (2.1–5.1)	52%	4.0 (2.4–6.7)	49%	2.1 (1.7–2.7)	57%	2.8 (2.1–3.8)

^1^ HR = hazard ratio; ^2^ CI = confidence interval.

**Table A2:** 5-year overall survival (OS) and cancer-specific survival (CSS) of patients operated on before and after 2011.

Characteristic	Overall Survival 5-Year	HR ^1^ (95% CI ^2^)	Cancer-Specific Survival 5-Year	HR ^1^ (95% CI ^2^)
Before 2011	62%	Reference	69%	Reference
After 2011	69%	0.97 (0.83–1.12)	77%	1.37 (1.15–1.64)
*p*-value	0.66		<0.05	

^1^ HR = hazard ratio; ^2^ CI = confidence interval.
