# Supplementary material for: Neoadjuvant Chemotherapy in Muscle-Invasive Bladder Cancer: A Nationwide Analysis of Eligibility, Utilization, and Outcomes
Source: Cancers (Basel). 2025 Feb 3;17(3):505. doi: 10.3390/cancers17030505 (PMC11816346; doi:10.3390/cancers17030505)
Supplement: Supplementary file 1 [file cancers-17-00505-s001.zip › cancers-3424022-supplementary.pdf]

Neoadjuvant Chemotherapy in Muscle-Invasive Bladder Cancer: A Nationwide Analysis of Eligibility, Utilization, and Outcomes

Ilkka Nikulainen [1,*], Antti P. Salminen [1], Mikael Högerman [1], Heikki Seikkula [2,†], Peter J. Boström [1,†] and The Finnish National Cystectomy Database Research Group [‡]

[1] Department of Urology, University of Turku and Turku University Hospital, 20521 Turku, Finland; antti.salminen@tyks.fi (A.P.S.); mikael.hogerman@tyks.fi (M.H.); peter.bostrom@tyks.fi (P.J.B.)

[2] Department of Surgery, Division of Urology, Central Hospital of Jyväskylä, Jyväskylä, Finland; heikki.seikkula@hyvaks.fi

* Correspondence: ilkka.nikulainen@tyks.fi; Tel.: +358-2-3137928; Fax: +358-2-3132284

† Shared last authorship.

‡ The Finnish National Cystectomy Database Research Group researchers is provided in the Supplementary Materials.

[‡]The Finnish National Cystectomy Database Research Group consists of the following researchers:

Ilkka Nikulainen, Department of Urology, University of Turku and Turku University Hospital, Turku, Finland

Antti P. Salminen, Department of Urology, University of Turku and Turku University Hospital, Turku, Finland

Mikael Högerman, Department of Urology, University of Turku and Turku University Hospital, Turku, Finland

Heikki Seikkula, Department of Surgery, Division of Urology, Central Hospital of Jyväskylä, Jyväskylä, Finland

Ileana Montoya Perez, Department of Computing, University of Turku, Turku, Finland

Jukka Sairanen, Department of Urology, University of Helsinki and Helsinki University Hospital, Helsinki, Finland

Ilmari Koskinen, Department of Urology, University of Helsinki and Helsinki University Hospital, Helsinki, Finland

Jussi Nikkola, Department of Urology, University of Tampere and TAYS Cancer Center, Tampere, Finland

Teemu J. Murtola, Department of Urology, University of Tampere and TAYS Cancer Center, Tampere, Finland

Markku H. Vaarala, Department of Urology, University of Oulu and Oulu University Hospital, Oulu, Finland

Senja Jousmäki, Department of Urology, University of Oulu and Oulu University Hospital, Oulu, Finland

Timo K. Nykopp, Department of Urology, University of Eastern Finland and Kuopio University Hospital, Kuopio, Finland

Taina Isotalo, Department of Surgery, Division of Urology, Central Hospital of Lahti, Lahti, Finland

Timo Marttila, Department of Surgery, Division of Urology, Central Hospital of Seinäjoki, Seinäjoki, Finland

Abdiwahid Alibeto, Department of Surgery, Division of Urology, Central Hospital of Mikkeli, Mikkeli, Finland

Marjo Seppänen, Department of Surgery, Division of Urology, Central Hospital of Pori, Pori, Finland

Christian Palmberg, Department of Surgery, Division of Urology, Central Hospital of Vaasa, Vaasa, Finland

Peter J. Boström, Department of Urology, University of Turku and Turku University Hospital, Turku, Finland
